# Supplementary material for: A whole-animal phenotypic drug screen identifies suppressors of atherogenic lipoproteins
Source: eLife. 2026 Aug 27;14:RP105314. doi: 10.7554/eLife.105314 (PMC13521566; doi:10.7554/eLife.105314)
Supplement: Supplementary file 2 [file elife-105314-code1.zip › JHDL_primary_result/Danthron/Danthron_JHDL_21_033_053_2019_10_11_12_17.pdf]

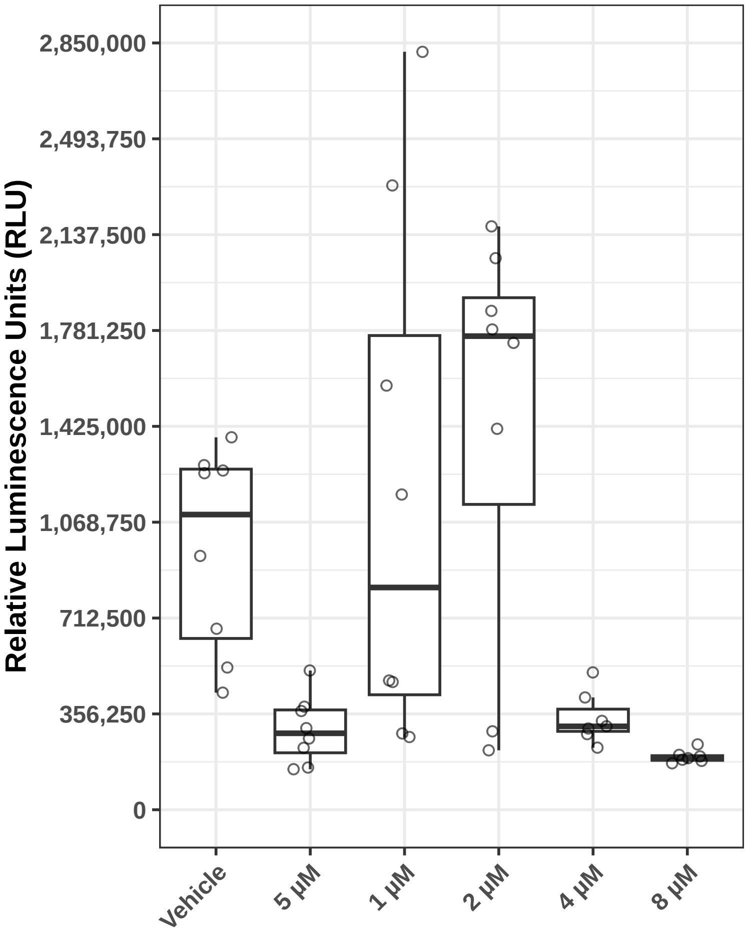
Relative Luminescence Units (RLU)
2,850,000
2,493,750
2,137,500
1,781,250
1,425,000
1,068,750
712,500
356,250
0
Vehicle
5 µM
1 µM
2 µM
4 µM
8 µM
